# Occurrence of 40 sanitary indicators in French digestates derived from different anaerobic digestion processes and raw organic wastes from agricultural and urban origin

**DOI:** 10.3389/fmicb.2024.1346715

**Published:** 2024-08-06

**Authors:** Caroline Wybraniec, Benoit Cournoyer, Cécile Moussard, Marion Beaupère, Léa Lusurier, Françoise Leriche, Karine Fayolle, Nicolas Sertillanges, Claire-Sophie Haudin, Sabine Houot, Dominique Patureau, Geneviève Gagne, Wessam Galia

**Affiliations:** ^1^Laboratoire d'Ecologie Microbienne, Research Group Bacterial Opportunistic Pathogens and Environment, Universite Claude Bernard Lyon, Villeurbanne, France; ^2^UMRF, Université Clermont Auvergne, INRAE, VetAgro Sup, Aurillac, France; ^3^INRAE, Univ. Montpellier, LBE, Narbonne, France; ^4^UMR ECOSYS, Université Paris-Saclay, INRA, AgroParisTech, Thiverval-Grignon, France

**Keywords:** organic waste, anaerobic digestion, virulence genes, antibiotic resistance genes, mobile genetic elements

## Abstract

This study investigated the sanitary quality of digestates resulting from the mesophilic anaerobic digestion (AD) of urban and agricultural organic wastes (OWs). 40 sanitary indicators, including pathogenic bacteria, antimicrobial resistance genes, virulence factor genes, and mobile genetic elements were evaluated using real-time PCR and/or droplet digital PCR. 13 polycyclic aromatic hydrocarbons (PAHs) and 13 pharmaceutical products (PHPs) were also measured. We assessed agricultural OWs from three treatment plants to study the effect of different AD processes (feeding mode, number of stages, pH), and used three laboratory-scale reactors to study the effect of different feed-supplies (inputs). The lab-scale reactors included: Lab1 fed with 97% activated sludge (urban waste) and 3% cow manure; Lab2 fed with 85% sludge-manure mixture supplemented with 15% wheat straw (WS); and Lab3 fed with 81% sludge-manure mixture, 15% WS, and 4% zeolite powder. Activated sludge favored the survival of the food-borne pathogens *Clostridium perfringens* and *Bacillus cereus*, carrying the toxin-encoding genes *cpe* and *ces, respectively.* Globally, the reactors fed with fecal matter supplemented with straw (Lab2) or with straw and zeolite (Lab3) had a higher hygienization efficiency than the reactor fed uniquely with fecal matter (Lab1). Three pathogenic bacteria (*Enterococcus faecalis*, *Enterococcus faecium*, and *Mycobacterium tuberculosis* complex), a beta-lactam resistance gene (*bla*_TEM_), and three mobile genetic elements (*intI1*, *intI2*, and IS*26*) were significantly decreased in Lab2 and Lab3. Moreover, the concentrations of 11 PAHs and 11 PHPs were significantly lower in Lab2 and Lab3 samples than in Lab1 samples. The high concentrations of micropollutants, such as triclosan, found in Lab1, could explain the lower hygienization efficiency of this reactor. Furthermore, the batch-fed reactor had a more efficient hygienization effect than the semi-continuous reactors, with complete removal of the *ybtA* gene, which is involved in the production of the siderophore yersiniabactin, and significant reduction of *intI2* and *tetO*. These data suggest that it is essential to control the level of chemical pollutants in raw OWs to optimize the sanitary quality of digestates, and that adding co-substrate, such as WS, may overcome the harmful effect of pollutants.

## 1 Introduction

According to the United Nations 2019 World Population Prospects report, the world population will reach 8.5 billion in 2030 and 10.9 billion in 2100. Therefore, the demand for food and energy, and the amount of generated organic wastes (OWs) will continue to increase (Hoornweg et al., 2013). Currently, the burning of fossil fuels to produce energy generates about two-thirds of global greenhouse gas emissions (Energy and climate change — European Environment Agency, 2017). Moreover, the natural decomposition of OWs releases greenhouse gases directly into the atmosphere, which contributes to global warming. Thus, a large-scale application of ecological methods to treat OWs and produce energy without generating greenhouse gases is needed. Anaerobic digestion (AD) of OWs, which produces renewable energy while reducing OW volume and methane emissions, is well-suited for this purpose (Energy and climate change — European Environment Agency, 2017). The resulting methane (CH_4_) can be converted into heat and electricity and the other main output (called digestate) can be used to amend agricultural land (Fagerström et al., 2018).

The AD process is driven by a diverse community of microbes (mainly bacteria and archaea). In the absence of oxygen, AD comprises a series of complex microbiological processes, including hydrolysis, acidogenesis, acetogenesis, and methanogenesis (Manyi-Loh et al., 2013; Abdelgadir et al., 2014). Many parameters, such as temperature, pH, volatile fatty acid (VFA) concentrations and ammonia concentrations, affect the ability of microorganisms to convert biomass to methane and impact the bacterial composition of the end products (Al Seadi et al., 2008). The bacterial community structure in digestates is also influenced by the origin of the raw OWs used in the digestion process. Aigle et al. (2021) showed that two different bacterial structures are present in raw OWs depending on their origin. In urban OWs (mainly activated sludge), *Proteobacteria* and *Bacteroidetes* were the most abundant phyla. In contrast, agricultural waste (mainly manure), contained *Firmicutes*, *Proteobacteria,* and *Bacteroidetes*. After AD, taxa from activated sludge increased among urban digestates. In contrast, taxa from raw agricultural OWs decreased in agricultural digestates (Aigle et al., 2021). This reshuffling of the bacterial community indicates that the origin of raw OWs plays an important role in the final bacterial content of digestates. However, the fecal matters frequently used as feedstock for AD (activated sludge and manure) are often contaminated with pathogenic bacteria (Hutchison et al., 2004; Sidhu and Toze, 2009). Therefore, it is important to understand the impact of the AD process on the fate of pathogenic bacteria in different OWs.

To address this question, many parameters, such as temperature, pH, and hydraulic retention time, have been investigated (Avadí et al., 2022). Temperature is crucial in AD processes because it affects the richness and diversity of the microbial community in digestates (Gou et al., 2014). Thermophilic digestion (55°C) is assumed to be more efficient than mesophilic digestion (35°C) in reducing pathogens such as *Escherichia coli* and *Salmonella* spp. (Smith et al., 2005). Also, with a high residence time, batch reactors ensure better sanitation than continuously fed processes (Jiang et al., 2018, 2020).

Beside pathogenic bacteria, other indicators such as helminth eggs can be used to evaluate the sanitary quality of digestates. Soil transmitted helminth infections are particularly widespread in tropical and sub-tropical countries where lack of drinking water and inadequate sanitary conditions favor their transmission (Riaz et al., 2020). Pathways of contamination may involve contaminated water, soil amended with contaminated digestate, and food grown on that soil (Nag et al., 2019). While full-scale thermophilic digestion seems to be sufficient to inactivate helminth eggs (Seruga et al., 2020), mesophilic digestion can be insufficient (Harroff et al., 2019). In some regions (e.g., Latin America), AD is often operated under mesophilic or psychrophilic conditions using, e.g., low-cost plastic tubular digesters (Garfí et al., 2016; Tavera-Ruiz et al., 2023). In these conditions, regardless of the feedstock (cow manure alone or in combination with cheese whey, pig manure, food waste), the AD process was insufficient to eliminate helminth eggs, and viable eggs were found in digestates at concentrations ranging from 5 to 50 per 4 g (Cucina et al., 2021; Parra-Orobio et al., 2021). However, Parra-Orobio et al. (2021) highlighted the effect of the anaerobic reactor configuration (single vs. two-stage configurations) on the parasitological properties of digestates. Interestingly, the best quality digestates were obtained using the anaerobic two-stage configuration. These studies highlight the importance of considering the configuration of anaerobic reactors but also the origin of organic waste and the region of their production in the choice of indicators that could be used to evaluate the health quality of digestates.

However, previous studies have shown conflicting results regarding the fate of pathogenic bacteria, even in similar digestion conditions. For example, Tápparo et al. (2020) showed that, during batch lab-scale mesophilic digestion using agricultural waste as feedstock, *E. coli* enumerated at 5–6 logs CFU/mL in raw waste and *Salmonella* at more than 6 logs CFU/mL. Both bacteria were removed in digestates within 8–10 days. However, in similar conditions, Alfa et al. (2014) found that 30 days were needed to reduce coliforms by 1 log and were insufficient to completely remove *Salmonella* from digestates. While *E. coli* and *Salmonella* are often used to estimate the hygienization effect of AD, the fate of other pathogens usually detected in raw OW has rarely been investigated in digestates. These pathogens include *Mycobacterium tuberculosis* complex (Santos et al., 2015)*, Yersinia enterocolitica*, *Yersinia pseudotuberculosis* (Falcão et al., 2004), and *Acinetobacter baumannii* (Seruga Music et al., 2017). In addition to their pathogenicity, these bacteria may carry antibiotic resistance genes (ARGs), conferring resistance to antibiotics commonly used to treat food-production animals, such as tetracycline and aminoglycosides (van Hoek et al., 2011). Microbial communities mainly acquire ARGs via horizontal transfer of mobile genetic elements (MGEs), such as plasmids, transposons, and integrons (von Wintersdorff et al., 2016). Several studies have reported that AD processes have a variable effect on MGEs and ARGs, depending on the feedstock and digestion conditions. For example, Zou et al. (2020) showed that the abundance of class 1 integrons (*intI1*) decreased by 0.9 log copies/g dry manure after 60 days of mesophilic digestion in batch lab-scale reactors. In contrast, in a feedstock composed of food waste and sewage sludge, *intI1* increased from 10^−4^ to 10^−3^ gene copies/16S rRNA copies after 33 days of mesophilic digestion (Zhang et al., 2016). Similarly, the total abundance of seven *tet* genes significantly increased after mesophilic AD of tetracycline-spiked livestock manure (Agga et al., 2020). However, the abundance of the resistome and MGEs was reduced in municipal sludge after thermophilic AD, even under high oxytetracycline concentrations (Tian et al., 2019).

Therefore, the fate and distribution of different pathogens, MGEs, and ARGs remain unclear, and more research is required to assess the biosafety of digestates. In this study, we assessed 77 sanitary indicators, including pathogenic bacterial species and ARGs, as well as new indicators of health hazards, such as virulence factors carried by MGEs. We first validated detection by real-time PCR and quantification by droplet digital PCR (ddPCR) for 40 and 31 sanitary indicators, respectively, in raw OWs and digestates. We then investigated the sanitary performance of AD according to the type of process (feeding mode, number of stages, temperature, pH) and the nature of the raw OWs (fecal matter with or without supplementation with straw and/or zeolite).

## 2 Materials and methods

### 2.1 Sample collection from full-scale anaerobic reactors

We selected three full-scale anaerobic OW treatment plants (Ter1, Ter2, and Ter3) with reactors representative of the most common treatment processes used in France. All reactors were fed with a similar OW of agricultural origin. The operating parameters of each reactor are described in Table 1. Three biological replicates including raw OWs (inputs) and digestates (outputs) were collected for each reactor, except Ter3, for which three input samples and five output samples were collected. All samples were transported on ice to the laboratory. For solid matrices (e.g., manure, silage), up to 24 sub-samples from different places were gathered to obtain a representative biological sample of 5 kg (dry mass). Each sample was mixed and then a homogenized 1 kg sample was collected. Liquid biological samples (e.g., slurry) were sampled from stirred tanks to obtain a representative 1 L sample. Samples were then mixed in a blender to obtain a composite sample of the raw OWs used to feed each reactor. Outputs were directly collected from the anaerobic reactors. All samples were stored at −20°C on the day of sampling.

**Table 1:** Operating parameters of anaerobic reactors sampled in this study.

Name	Reactor size	Temperature (°C)	Process-type	Feeding mode^a^	HTR (days)	pH	Inputs
Ter1	2,480 m^3^	44–45	Two-stage	Semi-continuous	50/stage	7.2–7.4	36% CM, 32% meslin, 16% various silage, 11% corn silage, 5% HM
Ter2	900 m^3^	39	Single stage	Semi-continuous	38	8.2–8.4	50% CS, 22% PS, 16% digestates, 5% CM,5% apple waste, 1% sorghum silage, 1% cow’s wasted ration
Ter3	600 m^3^	42–47		Batch	35–40	9.0	80% CM and 20% GW
Lab1	6 liters	37	Single stage	Semi-continuous	35	7.2–7.4	97% WWTP sludge, 3% CM
Lab2	6 liters	37	Single stage	Semi-continuous	56	7.2–7.4	85% of WWTP sludge and CM mixture (97:3 ratio), 15% WS
Lab3	6 liters	37	Single stage	Semi-continuous	56	7.2–7.4	81% of WWTP sludge and CM mixture (97:3 ratio), 15% WS, 4% zeolite clay powder

Three full-scale plants (Ter1, Ter2 and Ter3) were selected to study the effect of the key parameters of anaerobic digestion process (feeding mode, number of steps, temperature, pH) on the sanitary quality of digestate. The impact of the nature of raw wastes has been studied using 3 lab-scale reactors (Lab1, Lab2 and Lab3) where the anaerobic digestion process was similar, but the proportions of inputs have been varied (mainly fecal matters supplemented or not with straw or straw and zeolite). HTR, Hydraulic retention time; CM, Cow manure; HM, Horse manure; CS, Cow slurry; PS, Pig slurry; GW, Green waste; WS, Wheat straws.^a^In semi-continuous mode of feeding, raw organic wastes are added to the reactor once a day with a partial withdrawal of digestates.

### 2.2 Sample collection from lab-scale anaerobic reactors

The digestion experiment was conducted in three lab-scale (6 L) stainless steel reactors (Lab1, Lab2, and Lab3). Digested secondary sludge from a wastewater treatment plant (WWTP) in the center of France (285,000 population equivalents) was used to launch the AD process before starting the experiments. The reactors were continuously mixed at 90 rpm during at least three hydraulic retention time cycles at a constant 37.5°C maintained by hot water circulation (Table 1 and Supplementary Table S1). Lab1 was fed with 97% activated sludge (Limoges, France) considered as urban waste and 3% cow manure (farm in Chateauroux, France). Lab2 was fed with 85% of the same mixture, supplemented with 15% commercial wheat straw (WS). Lab3 was fed with 81% of the same mixture, supplemented with 15% WS and 4% zeolite powder (ZP) (aluminosilicate mineral, Merck, France). A frozen stock of WWTP activated sludge, cow manure, and wheat straws was kept at 4°C and used to manually feed the reactors each day to produce an equal amount of discharged digestates. Lab1 was run for 18 weeks with an organic loading rate of 2.8 g volatile solids (*VS*)/L/day. Lab2 and Lab3 were run for 38 weeks with the same organic loading rate for 12 weeks (2.8 *VS*/L/day), then, due to VFA accumulation, the organic loading rate was then lowered to 1.3 g *VS*/L/day until the end of the experiment (Supplementary Table S2). Analysis of volatile solids/dry matter (*VS*/DM) was continuously performed to assess matter reduction during treatment. Gas flow and composition and pH were also monitored (Aemig et al., 2019). After manual collection, digestates were kept at 4°C until mixing to obtain one sample per week. For Lab2 and Lab3, biological samples of inputs were collected on weeks 27, 35, and 40. Biological samples of digestates were collected on weeks 26, 34, and 38. For Lab1, inputs and outputs were collected on weeks 10, 14, and 17 (Supplementary Table S1). All samples were stored at −20°C.

### 2.3 Analysis of volatile solids/dry matter

Dry matter and volatile solids contents were measured in triplicate by calculating the mass difference after 24 h at 105°C and after an additional 2 h at 550°C. The remaining ashes were considered to be mineral matter (MM) (Supplementary Tables S2, S3). The pH was directly measured on liquid samples or according to ISO 10390.

### 2.4 Analysis of pharmaceutical products and polycyclic aromatic hydrocarbons

Analysis of pharmaceutical products (PHPs) and polycyclic aromatic hydrocarbons (PAHs) were performed on samples from lab-scale anaerobic reactors. PAHs were extracted from a 1 g subsample according to Trably et al. (2004) and Mailler et al. (2017). Control matrices with known PAH concentrations were extracted in parallel. Solvent extracts were then evaporated and dissolved in 100% acetonitrile. Each extract was analyzed using a Waters Alliance 2,695 High-Performance-Liquid-Chromatography system coupled to a fluorescence detector as described by Sertillanges et al. (2020). Pharmaceutical products and biocides were extracted using the method described by Ferhi et al. (2016). Ultrasonic extraction was performed only on dry matter. Sludge was extracted in an acetonitrile/McIlvaine buffer/EDTA mix. After purification, pharmaceutical products and biocides were quantified in samples using high-performance liquid chromatography and mass spectrometry with a Xevo TQD Triple Quadrupole mass spectrometer (Waters). In order to assess both purification/extraction losses and potential matrix effects in mass spectrometry (MS), the compound concentrations were determined by internal quantification-isotope dilution as described by Sertillanges et al. (2020). Limits of quantification (LOQs) for each compound were estimated as the concentration corresponding to a signal-to-noise ratio of 10. Limits of detection (LODs) were considered equivalent to LOQ/3. Concentrations of all organic micropollutants are expressed on a DM basis.

### 2.5 DNA extraction

Three independent DNA extractions per sample were performed using the FastDNA Spin Kit For Soil (MP Biomedicals), following the manufacturer’s instructions. DNA quantity and quality were measured using a NanoDrop One (Eurobio Scientific) and then kept at −20°C until further analysis. All DNA used in this project was tested for the presence of PCR inhibitors as described in Voisin et al. (2020). Briefly, 2 μL of plasmid carrying the *intI2* gene (10^5^ copies per μl) was added to 16 μL of Brilliant II SYBR® Green QPCR Master Mix (Agilent) and 2 μL of target DNA. The number of cycles required to observe a significant signal was compared with that of samples containing only the plasmid with the *intI2* gene and the real-time PCR (RT PCR) mix. Samples were processed in the same way as for real-time PCR (see section 2.6). When a higher number of cycles was required to obtain a signal, indicating potential PCR inhibition, DNA samples were diluted by 5- or 10-fold and another run was realized to confirm the absence of PCR inhibition after dilution.

### 2.6 Real-time PCR

Real-time PCR was used to detect specific DNA fragments from the targeted sanitary indicators in inputs and outputs from anaerobic reactors. The 20 μL real-time PCR reaction mix contained a final concentration of 500 nM of forward and reverse primers, 10 μL of Brilliant II SYBR^®^ Green QPCR Master Mix (Agilent), and 2 μL of target DNA. Amplification was run on a CFX96™ Real-Time System (BioRad) thermal cycler. The steps for real-time PCR amplification were as follows: 1 cycle at 95°C for 10 min; followed by 40 cycles of 95°C for 15 s and an amplification step for 30 s at optimal hybridization temperature for each primer pair (Supplementary Table S4). Melting curve analysis was conducted between 65°C and 95°C with 0.5°C increments and 5 s/increment. Negative controls were performed in triplicate for each run, using H_2_O instead of DNA. A list of the primers used, the expected size of each amplified DNA fragment, and hybridization temperatures are shown in Table 2 and Supplementary Table S4. All primers were synthesized by Invitrogen (Paris, France).

**Table 2:** Specific detection by real-time PCR of 40 sanitary indicators of public health concern in organic waste bacterial communities from agricultural and urban origin.

Sanitary indicator		Target gene	Obtained MT (theoretical MT) °C	Product (bp)	Reference sequence ID (identity % with reference sequence)
*B. cereus* group		*gyrB*	77.0 (77.5)	221	CP054800.1 (73)
*Bacteroides*		16S rDNA	77.0 (77.0)	81	LR999587.1 (100)
*C. burnetii*		*ompA*	81.5 (81.5)	82	CP103435.1 (87)
*C. perfringens*		*plc*	76.0 (NI)	219	CP075979.1 (96)
*E. faecium*		*ddl*	76.5 (75.0)	140	CP038996.1 (97)
*E. faecalis*		*ddl*	74.0 (74.0)	74	CP110289.1 (65)
ETEC		*gspD*	79.0 (79.0)	55	CP104442.1 (73)
*K. pneumoniae*		*Kpn gltA*	77.5 (77.5)	68	CP114391.1 (86)
*L. monocytogenes*		*hlyA*	75.0 (NI)	112	CP032669.1 (93)
*M. tuberculosis complex*		*IS6110* ^a^	89.5 (89.0)	245	CP095023.1 (77)
*P. aeruginosa*		*ecfX*	82.5 (82.5)	81	CP104565.1 (95)
*S. agalactiae*		*cpsE*	73.0 (73.0)	85	JN384113.1 (78)
*Y. enterocolitica*		*ystB* ^ab^	79.5 (79.5)	146	CP107100.1 (82)
*C. perfringens* enterotoxin		*cpe*	74.0 (76.0)	112	MH900564.1 (90)
*B. cereus* emetic toxin		*ces*	76.5 (79.0)	176	DQ360825.1 (84)
Loci encoding the siderophore yersiniabactin		*irp2*	84.0 (NI)	280	CP009712.1 (90)
		*ybtA*	85.5 (85.5)	233	U50452.1 (91)
Class 1	Integron integrase	*intI1*	87.0 (87.0)	217	NC_024987.1 (94)
Class 2		*intI2*	80.0–80.5 (80.0)	195	CP044485.1 (99)
IS*26*	Insertion sequence	IS*26*	85.0 (NI)	275	AP022548.1 (94)
IS*Ecp1*		IS*Ecp1*	78.0 (78.0)	273	AP022255.1 (100)
IncFIC plasmid	Region related to replication	*IncFICrep*	81.0 (81.0)	281	MK492260.1 (93)
IncN plasmid		IncN *rep*^ab^	82.0 (NI)	164	CP117743.1 (90)
IncP plasmid		IncP *oriT*	83.0–83.5 (83.5)	110	MT868884.1 (76)
IncQ plasmid		IncQ *oriT*	84.0 (84.0)	191	CP053727.1 (90)
Subunit of AcrA-AcrB-TolC multidrug efflux complex		*acrA*	81.0 (81.0)	70	NC000913.3 (83)
		*acrB*	80.5 (80.5)	74	CP117717.1 (87)
Subunit of many multidrug efflux complexes in Gram^−^ bacteria		*tolC*	77.0 (77.0)	64	NC000913.3 (76)
Subunit of qac multidrug efflux pump		*qacH/E*	79.5 (79.5)	115	AY816216.1 (68)
Efflux pump conferring resistance to fluoroquinolone		*oqxA*	85.0–85.5 (85.5)	199	CP114853.1 (84)
Extended-spectrum class A beta-lactamase		*bla_CTX-M_*	81.5–82.0 (81.5)	103	KU760765.1(89)
		*bla_TEM_*	79.5 (79.5)	113	CP114854.1 (90)
		*bla_SHV_*	83.5 (83.5)	110	AF226622.1 (97)
23S rRNA (adenine(2058)-N(6))-methyltransferase		*ermB*	79.0 (79.0)	135	MN461246.1 (90)
Tetracycline efflux MFS transporter		*tetA*	81.0 (81.0)	71	CP080428.1 (95)
Tetracycline resistance-associated transcriptional repressor		*tetC*	76.0 (77.0)	105	CP109602.1 (100)
Tetracycline resistance ribosomal protection		*tetO*	77.5 (77.5)	104	CP113954.1 (92)
Sulfonamide-resistant dihydropteroate synthase		*sul1*	82.0 (82.0)	102	NC_019368.1(89)
		*sul2*	84.0 (84.0)	105	AP026908.1 (98)
Vancomycin-resistant enterococci		*vanA* ^ab^	81.0 (81.5)	231	CP114619.1 (94)

^a^Amplification of the correct product in addition to several nonspecific products were observed in agricultural wastes. ^b^Amplification of the correct product in addition to several nonspecific products were observed in urban wastes. NI, Not indicated; MT, melting temperature; VF, Virulence factors; ARG, Antibiotic resistance genes; MGE, Mobile genetic elements. Bacterial genera or species analyzed in this study: *Bacillus cereus* group (*B. cereus* group); *Bacteroides* (16S rDNA); *Coxiella burnetii (C. burnetiid); Clostridium perfringens* (*C. perfringens*); *Enterococcus faecium* (*E. faecium*); *Enterococcus faecalis* (*E. faecalis*); Enterotoxigenic *Escherichia coli* (ETEC); *Klebsiella pneumoniae* (*K. pneumoniae*); *Listeria monocytogenes* (*L. monocytogenes*)*; Mycobacterium tuberculosis* complex (*M. tuberculosis* complex); *Pseudomonas aeruginosa* (*P. aeruginosa*); and *Streptococcus agalactiae* (*S. agalactiae*); *Yersinia enterocolitica* (*Y. enterocolitica*).

### 2.7 Sequencing of PCR products and validation of primer specificity

The identities of amplified gene targets were evaluated through analysis of the melting curve. For each run, the size of the amplified DNA fragment was estimated after electrophoresis at 6 v/cm using a TBE buffer [89 mM Tris-borate, 89 mM boric acid, and 2 mM EDTA (pH 8.0)] through a 2% (w/v) agarose gel. A 50 bp DNA ladder from Thermo Scientific was used as a molecular weight marker. Gels were imaged and analyzed using Molecular Imager Gel Doc XR+ (BioRad, France). Then, at least one replicate from the positive sample was submitted for Sanger sequencing with the forward and/or the reverse primer (GenoScreen, France). Our samples were only considered positive for targeted genes if they demonstrated all of the following: (1) no-template controls performed as expected; (2) the DNA sample displayed an amplification curve; (3) the DNA sample displayed the expected melting curve peak; (4) the DNA sample displayed the expected DNA size on agarose gel, and (5) Sanger sequencing produced a clean chromatogram that matched the expected sequence for each gene with a high similarity percentage. To estimate the similarity percentage between the amplified sequence and the target sequence, sequenced DNA was compared with reference sequences of each targeted gene obtained from the NCBI database (Table 2). For each sample that met all of these criteria, the corresponding DNA was considered positive and specific for the prevalence real-time PCR assay (Supplementary Table S4). If any of these criteria was not met, the sample was included in prevalence analysis but was removed from the ddPCR quantification assay (Table 2).

### 2.8 Droplet digital PCR

Absolute quantification of targeted genes was performed in a QX200 Droplet Digital PCR System (Bio-Rad Laboratories, CA), according to the manufacturer’s instructions (Hindson et al., 2011). Each test was prepared in 22 μL of the reaction mixture, which contained 11 μL QX200 EvaGreen ddPCR Supermix (2X), 1.1 μL forward primer (2 μM), 1.1 μL reverse primer (2 μM), 4 μL target DNA, and 4.8 μL H_2_O. Negative controls without DNA template were performed in duplicate for each ddPCR run. For microdroplet generation, 70 μL droplet generation oil and 20 μL mixture were added to the DG8™ cartridge (Bio-Rad), then loaded into a QX200 Droplet Generator (Bio-Rad). Then, microdroplets were transferred to a ddPCR 96-well plate and heat-sealed with an aluminum film. PCR was performed in a T100 (BioRad) thermal cycler under the following conditions: 95°C for 5 min; followed by 40 cycles of 95°C for 30 s and hybridization/elongation temperature for 60 s. The post-cycling protocol (signal stabilization) was 4°C for 5 min and 90°C for 5 min. After amplification, droplets were kept at 4°C. Finally, the fluorescence signal in each microdroplet was quantified by the QX200 Droplet Reader (BioRad) and analyzed with QuantaSoft software Version 1.7.4 (BioRad) (Cheng et al., 2019; Zeng et al., 2020).

### 2.9 Statistical analysis

Statistical analysis was performed using Rstudio software (R version 4.1.3). Principal component analysis was performed using FactoMineR v2.7 package (Lê et al., 2008) and missing values were imputed using the missMDA v1.18 package (Josse and Husson, 2016). Kruskal-Wallis non-parametric tests with Dunn’s post-hoc tests were performed on PHP and PAH concentrations. Fisher tests were performed on the prevalence measurements of the sanitary indicators to compare the inputs and outputs. Values of absolute abundance equal to 0 were replaced by 0.1 and data were log10 transformed before statistical analysis. To test the influence of the technological process or the nature of raw waste on the absolute abundance of sanitary indicators, generalized linear models (GLMs) were fitted with a gamma likelihood [formula: Absolute abundance ~ Process or nature of raw waste * Nature of the sample (input or output)]. Pairwise comparisons between inputs and outputs of each reactor were performed using the estimated marginal means and *p*-values were adjusted using Bonferroni’s Holm method with emmeans_test function from rstatix package. For statistical analysis, *p* < 0.05 was considered to be statistically significant.

## 3 Results

### 3.1 Physico-chemical characterization of raw organic wastes and digestates

Three full-scale reactors (Ter1, Ter2, Ter3) representative of the most common AD processes used in France were identified in the Auvergne Rhône-Alpes region. Samples were collected from these reactors to investigate the effect of different AD processes. The impact of the type of OW input was studied using three lab-scale reactors (Lab1, Lab2, Lab3), in which the AD process was fixed and the proportions of inputs were varied (for more details, see Table 1). The key parameters of each reactor and the physicochemical characteristics of the OWs were measured (Table 1 and Supplementary Tables S2, S3). The average pH was 7.3 for the Ter1, Lab1, Lab2, and Lab3 reactors. The Ter2 and Ter3 reactors had a more basic pH (8.3 for Ter2 and 9.0 for Ter3). OWs from Ter1 and Ter2 had similar dry matter content (20.8 and 17.1%, respectively), while the input from Ter3 had a higher dry matter content (26.9%). Organic matter represented between 69 and 76% of the dry matter in the inputs of the three full-scale reactors (Ter1, Ter2, and Ter3). A significant decrease (16.2%) in the amount of organic matter in the outputs, compared with the inputs, was observed in Ter3 (batch reactor). This decrease was significantly lower for the semi-continuous reactors Ter1 and Ter2 (0.8 and 3.9%, respectively). This reduction can be explained by the partial transformation of organic matter into biogas (in the form of CO_2_ and CH_4_) during the AD process. For the laboratory-scale reactors, the percentage of dry matter was lower, ranging from 6.7% (Lab2, with added straw), to 8.2% (Lab1) and 8.3% (Lab3, with added straw and zeolite powder). The amount of organic matter in the dry matter was between 72.5% (Lab3) and 85.0% (Lab2). The decrease in organic matter after AD was higher in the Lab1 reactor (8.2%) than in the Lab2 and Lab3 reactors (3.8 and 1.3%, respectively).

### 3.2 Organic micropollutants in raw organic wastes and digestates

**Figure 1:**
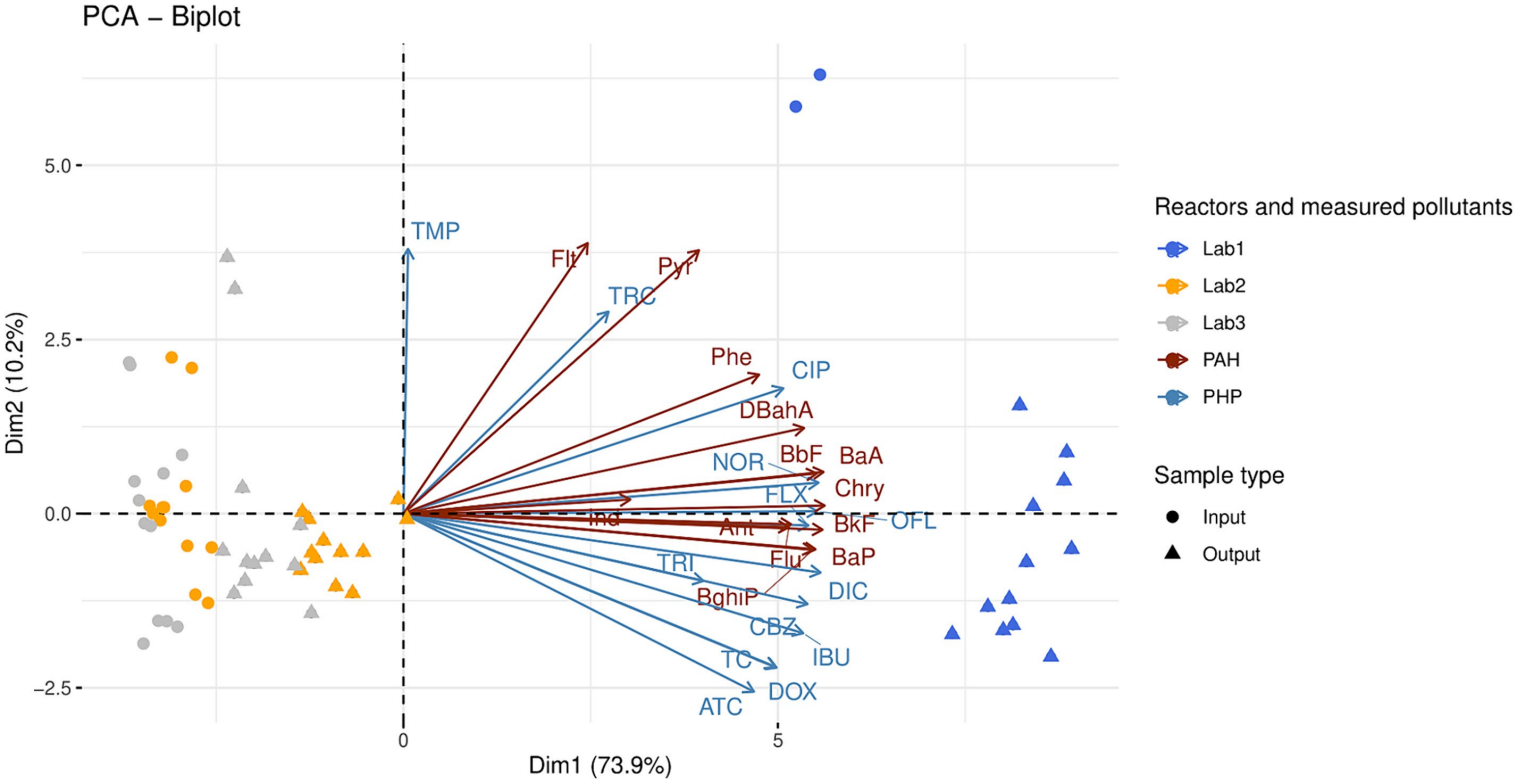
Principal component analysis of 13 polycyclic aromatic hydrocarbons (PAH) and 13 pharmaceutical products (PHP) measured in the raw organic wastes (input) and their corresponding digestates (output). The anaerobic digestion process was similar in the three reactors (Lab1, Lab2, and Lab3) but the proportions of inputs varied (see Table 1 for more information on operating parameters of Lab1, Lab2, and Lab3). Kruskal-Wallis non-parametric tests with Dunn’s post-hoc tests were performed to further investigate the observed distributions (see text and Supplementary Tables S5, S6). Abbreviations of polycyclic aromatic hydrocarbons: Anthracene (Ant); Benzo.a.Anthracene (BaA); Benzo.a.Pyrene (BaP); Benzo.b.Fluoranthene (BbF); Benzo.g.h.i.Perylene (BghiP); Benzo.k.Fluoranthene (BkF); Chrysene (Chry); DiBenzo.a.h.Anthracene (DBahA); Fluoranthene (Flt); Fluorene (Flu); Indeno.1.2.3.c.d.Pyrene (Ind); Phenanthrene (Phe); Pyrene (Pyr). Abbreviations of pharmaceutical products: Norfloxacin (NOR); Ofloxacin (OFL); Ciproflaxin (CIP); Doxycycline (DOX); Tetracycline (TC); Anhydrotetracycline (ATC); Trimethoprim (TMP); Fluoxetine (FLX); Carbamazepine (CBZ); Diclofenac (DIC); Ibuprofen (IBU); Triclosan (TRI); Triclocarban (TRC).

Thirteen PHPs, including seven antibiotics (three from the fluoroquinolone-quinolone family, three from the tetracycline family, and trimethoprim), one antidepressant (fluoxetine), one antiepileptic (carbamazepine), two anti-inflammatories (diclofenac and ibuprofen), and two antibacterial molecules (triclosan and triclocarban), were measured in raw OWs and digestates from the laboratory reactors Lab1, Lab2, and Lab3 (Supplementary Table S5). In addition to the PHPs, thirteen PAHs were also identified (Supplementary Table S6). Principal component analyses (PCAs) were performed on both types of pollutant (PAHs and PHPs, Figure 1). The first two axes of these PCAs explain a significant portion of the variability (84.1%) among reactors. The first component alone explains 73.9% of this variability and is correlated with PHP and PAH concentrations. Lab1 samples (raw OWs and digestates) were differentiated from Lab2 and Lab3 samples by higher concentrations of both pollutants. Kruskal-Wallis non-parametric tests with Dunn’s post-hoc tests were performed to further investigate the observed distributions (Supplementary Tables S5, S6).

The Lab1 samples (digested or not) could be differentiated from both Lab2 and Lab3 samples by a set of pollutants, including 11 PHPs (norfloxacin, ofloxacin, ciprofloxacin, doxycycline, tetracycline, anhydrotetracycline, fluoxetine, carbamazepine, diclofenac, ibuprofen, and triclosan), and 11 PAHs (anthracene, benzo(a)anthracene, benzo(b)fluoranthene, benzo(ghi)perylene, benzo(k)fluoranthene, chrysene, dibenzo(ah)anthracene, fluorene, phenanthrene, and pyrene, KW– Dunn’s *post hoc* tests; *p* < 0.05, Supplementary Tables S5, S6). Interestingly, AD had a moderate impact on the concentration of norfloxacin in Lab1 and on the concentration of trimethoprim in both Lab1 and Lab2. The concentration of the other PHPs was not significantly impacted by this process. Therefore, their concentrations in raw OW samples were compared between the three reactors Lab1, Lab2, and Lab3 (Supplementary Table S5). Ibuprofen and triclosan were only detected in Lab1 inputs, at 43.4 ± 3.3 μg/kg and 2100.0 ± 707.1 μg/kg, respectively. Moreover, compared with Lab2, Lab1 showed significantly higher concentrations (*p* < 0.05) of two other PHPs: norfloxacin (1318.6 ± 36.5 μg/kg in Lab1 *vs* 132.4 ± 20.0 μg/kg in Lab2) and fluoxetine (51.2 ± 5.7 μg/kg in Lab1 *vs* 17.3 ± 6.9 μg/kg in Lab2). Compared with Lab3, Lab1 also showed significantly higher concentrations of ciprofloxacin (2645.7 ± 205.9 μg/kg in Lab1 *vs* 775.7 ± 85.7 μg/kg in Lab3), anhydrotetracycline (41.9 ± 16.9 μg/kg in Lab1 *vs* 0.0 μg/kg in Lab3), and diclofenac (123.9 ± 9.7 μg/kg in Lab1 *vs* 38.7 ± 10.5 μg/kg in Lab3). Finally, ofloxacin, doxycycline, tetracycline, trimethoprim, and carbamazepine were significantly higher in Lab1 and Lab2 inputs, compared with Lab3.

PAH concentrations were also compared between the three reactors Lab1, Lab2, and Lab3. Generally, the overall PAH concentration (sum of the 13 PAHs measured in this study) was higher in raw OWs used to feed Lab1 (1274.9 ± 11.7 μg/kg) than in raw OWs supplemented with straw and used to feed Lab2 (426.9 ± 63.3 μg/kg) or raw OWs supplemented with straw and zeolite and used to feed Lab3 (422.4 ± 58.8 μg/kg). After AD, the PAH concentrations were unchanged in Lab1 outputs (1297.7 ± 137.8 μg/kg), but were increased in Lab2 (678.7 ± 58.9 μg/kg, *p* < 0.01) and Lab3 (607.5 ± 103.0 μg/kg, ns) outputs, compared with PAH concentrations in the respective raw OW of each reactor (Supplementary Table S6).

### 3.3 Impact of anaerobic digestion on sanitary indicators

#### 3.3.1 Specific amplification of sanitary indicators in organic waste bacterial communities from agricultural and urban origin

We validated specific real-time PCR amplification of 40 of the 77 sanitary indicators tested in raw OWs, and digestates, from agricultural and urban origin (Table 2 and Supplementary Table S4). These PCR assays targeted: (1) bacterial pathogens; (2) virulence genes; (3) MGEs; and (4) ARGs. Four sanitary indicators (the EHEC hemolysin operon, the insertion sequence IS*6*/*257*, *A. baumanii,* and *Salmonella hadar*) were not detected in the OW samples (*n* = 38) analyzed in this study (Supplementary Table S4). Targeted genes from 13 pathogenic species/genera, four virulence factors, eight MGEs, and 15 ARGs were specifically amplified in at least one sample (Table 2). Their specific amplification was evaluated by melt curve analysis and amplicon length estimated on agarose gel electrophoresis. Moreover, the specificity of each target gene was validated by nucleotide sequence analysis of at least one amplified product. Obtained sequences were closely related (more than 68%) to accurate annotated bacterial genes present in GenBank as verified by Clustal Omega multiple sequence alignments (Table 2).

Only primer pairs that produced a unique product verified by real-time PCR were used in ddPCR assays to quantify the target gene. This is because fluorescence linked to the amplification of nonspecific products can be identified in real-time PCR, but not in ddPCR. Thus, *Y. enterocolitica*, IncN, and *vanA* could not be analyzed by ddPCR because we observed real-time PCR amplification of several products in samples from agricultural and urban origin (Table 2). Furthermore, as *acrA*, *acrB*, and *tolC* genes are part of the same multidrug efflux complex, only *tolC* subunit primers were used to investigate this mechanism by ddPCR. Of the three genes implicated in tetracycline resistance *tetA* (encoding for efflux protein), *tetO* (encoding for ribosomal protection protein), and *tetC* (resistance-associated transcriptional repressor) only *tetA* and *tetO* were investigated by ddPCR.

#### 3.3.2 Prevalence of sanitary indicators in raw organic wastes and digestates

**Figure 2:**
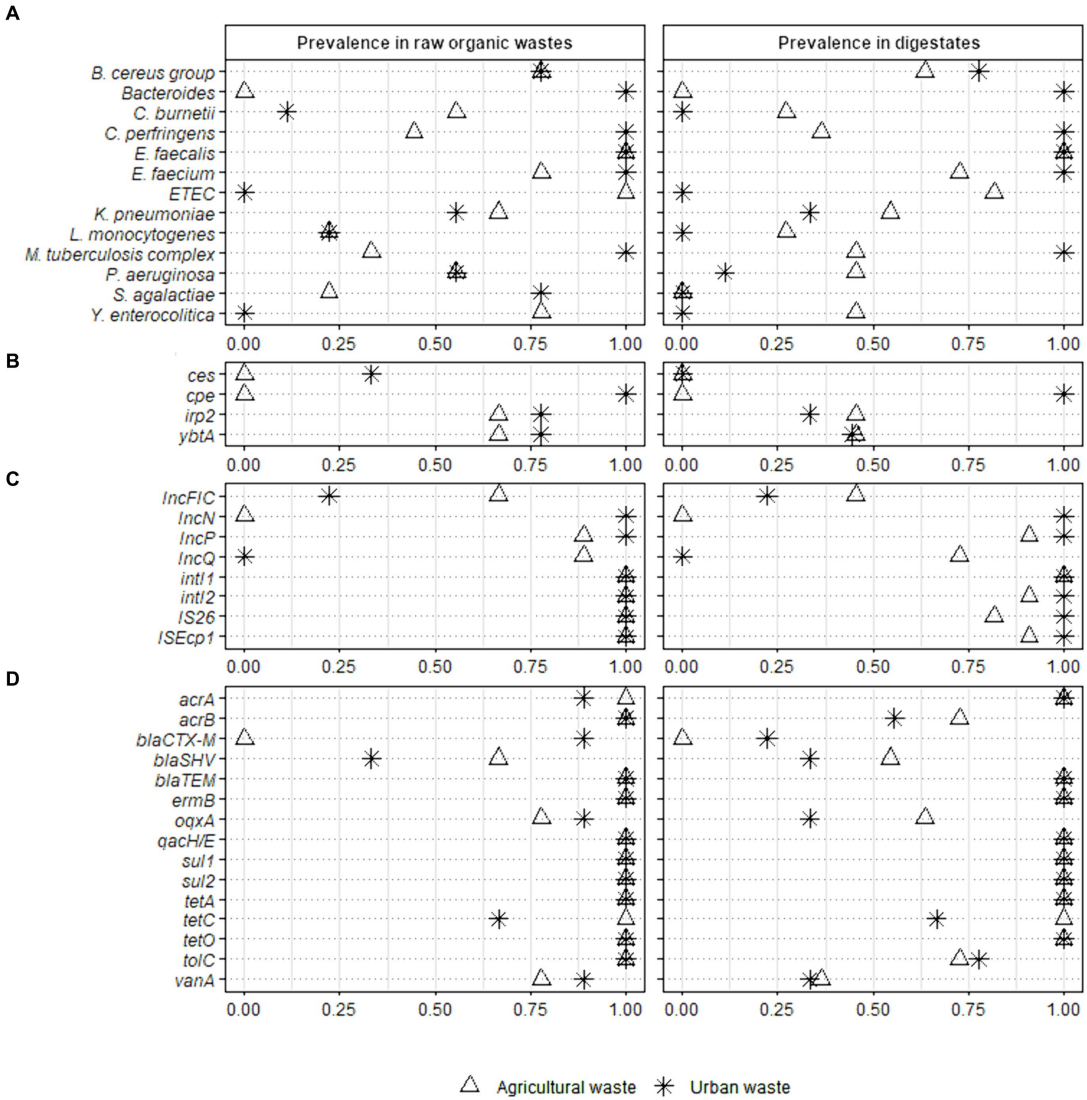
Prevalence of 40 sanitary indicators of public health concern in raw organic wastes from agricultural and urban origin and their derivative digestates. Full-scale reactors (Ter1, Ter2, and Ter3) were used to study the effect of the key parameters of anaerobic digestion while lab scale reactors (Lab1, Lab2, and Lab3) were used to investigate the impact of the nature of raw wastes on the sanitary quality of digestates. Fisher test was performed on raw organic wastes from agricultural (*n* = 9) and urban (*n* = 9) origin versus their respective digestates (11 and 9 samples analyzed from agricultural and urban origin, respectively) (* *p* < 0.05; ** *p* < 0.01). The sanitary indicators were classified into 4 categories: **(A)** bacterial genera or species, **(B)** virulence factors genes, **(C)** mobile genetic elements, and **(D)** antimicrobial resistance genes (see Table 2 for more information on these indicators genes).

We compared the prevalence of the 40 validated sanitary indicators in raw OWs and digestates after AD (Figure 2 and Supplementary Table S7).

##### 3.3.2.1 Sanitary indictors in raw organic wastes

The 40 indicators were classified into three categories according to their prevalence in raw OW samples (*n* = 18): (i) high prevalence (found in more than 75% of samples); (ii) medium prevalence (found in 25 to 75% of samples); (iii) low prevalence (present in less than 25% of analyzed samples), or absence (Figure 2 and Supplementary Table S7). The *E. faecalis*, *E. faecium*, and *B. cereus* group had a high prevalence (1.00, 95% CI 0.82–1.00; 0.89, 95% CI 0.67–0.97; and 0.78, 95% CI 0.55–0.91, respectively) in all raw OW samples (Supplementary Table S7). In contrast both *Y. enterocolitica* and enterotoxigenic *E. coli* (ETEC) were found only in raw agricultural wastes (0.78, 95% CI 0.45–0.94 and 1.00, 95% CI 0.70–1.00, respectively), and were not detected in wastes mainly composed of WWTP sludge (Figure 2 and Supplementary Table S7). Also, 25 to 75% of raw agricultural waste samples (*n* = 9) were positive for *Klebsiella pneumoniae* (0.67, 95% CI 0.35–0.88), *Clostridium perfringens* (0.44, 95% CI 0.19–0.73), *Pseudomonas aeruginosa* (0.56, 95% CI 0.27–0.81), the *M. tuberculosis* complex (0.33, 95% CI 0.12–0.65), and *Coxiella burnetii* (0.56, 95% CI 0.27–0.81, Figure 2 and Supplementary Table S7). *Listeria monocytogenes* and *Streptococcus agalactiae* were found with a low prevalence (<25%), while *Bacteroides* were not detected in the raw agricultural samples from the three full-scale reactors (Figure 2).

In the waste mainly composed of WWTP sludge, >75% of samples (*n* = 9) were positive for *S. agalactiae* (0.78, 95% CI 0.45–0.94), *C. perfringens* (1.00, 95% CI 0.70–1.00), *Bacteroides* (1.00, 95% CI 0.70–1.00), and the *M. tuberculosis* complex (1.00, 95% CI 0.70–1.00, Figure 2). Both *K. pneumoniae* and *P. aeruginosa* were found with a medium prevalence (0.56, 95% CI 0.27–0.81), *L. monocytogenes* and *C. burnetii* were weakly present (<25%), while *Y. enterocolitica* and ETEC were not detected (Figure 2 and Supplementary Table S7).

The *irp2* gene, encoding an iron regulatory protein involved in biosynthesis of the siderophore yersiniabactin, and *ybtA*, encoding a transcriptional regulator of the same gene cluster, were both found with a medium prevalence (0.67, 95% CI 0.35–0.88) in raw agricultural OWs and a high prevalence (0.78, 95% CI 0.45–0.94) in samples mainly composed of WWTP sludge (Figure 2). The other two virulence genes, *ces*, which is involved in cereulide biosynthesis and is produced by the *B. cereus* group, and *cpe*, which encodes an enterotoxin produced by *C. perfringens*, were not found in OWs from agricultural origin (*n* = 9). In contrast, the *cpe* and *ces* genes were detected with a high (1.00, 95% CI 0.70–1.00) and medium (0.33, 95% CI 0.12–0.65) prevalence, respectively (Figure 2 and Supplementary Table S7), in the OWs composed mainly of WWTP sludge (*n* = 9).

Interestingly, all raw OW samples (*n* = 18) were positive (1.00, 95% CI 0.82–1.00) for both integrons *intI1* and *intI2*, and the insertional sequences IS*26* and IS*Ecp1* (Figure 2 and Supplementary Table S7). Several broad-host-range plasmids of the IncP, IncQ, IncN, and IncFIC groups were also present in the raw OWs. IncP-specific sequence was found with a high prevalence (0.94, 95% CI 0.74–0.99) in raw OWs regardless of their origin, while the prevalence of the IncQ- and IncN-specific sequences varied with the origin of the OW sample (Figure 2 and Supplementary Table S7). The IncQ plasmids were detected with a high prevalence (0.89, 95% CI 0.57–0.98) in waste of agricultural origin, while the IncN plasmids were detected mainly in waste composed principally of WWTP sludge (1.00, 95% CI 0.70–1.00). Interestingly, IncN plasmids were not detected in agricultural waste and IncQ plasmids were not detected in waste composed mainly of WWTP sludge (Figure 2). The IncFIC plasmids were found with a medium prevalence (0.67, 95% CI 0.35–0.88) in agricultural waste and a low prevalence (0.22, 95% CI 0.06–0.55) in waste composed mainly of WWTP sludge.

We then assessed the prevalence of 15 ARGs. The multidrug resistance genes *acrB*, *tolC*, and *qacH/E*, the sulfonamide resistance genes *sul1* and *sul2*, the tetracycline resistance genes *tetA* and *tetO*, and the macrolide resistance gene *ermB* were found in all (18/18) raw OW samples (1.00, 95% CI 0.82–1.00, Figure 2 and Supplementary Table S7). We also found a high prevalence of *acrA* (0.94, 95% CI 0.74–0.99), *tetC* (0.83, 95% CI 0.61–0.94), the quinolone resistance gene *oqxA* (0.83, 95% CI 0.61–0.94), and the vancomycin resistance gene *vanA* (0.83, 95% CI 0.61–0.94) in raw OWs regardless of their origin. Similarly, the beta-lactam resistance gene, *bla*_TEM_ was detected with a high prevalence (1.00, 95% CI 0.82–1.00) in both agricultural (*n* = 9) and urban OWs (*n* = 9) (Figure 2 and Supplementary Table S7). The *bla*_CTX-M_ gene was detected mainly in waste composed of WWTP sludge (0.89, 95% CI 0.57–0.98) and was not found in waste of agricultural origin. The *bla_SHV_* gene was found with a medium prevalence in both agricultural (0.67, 95% CI 0.35–0.88) and urban wastes (0.33, 95% CI 0.12–0.65, Figure 2).

##### 3.3.2.2 Effect of anaerobic digestion on the prevalence of sanitary indicators

The prevalence of only one pathogenic bacterial species, *S. agalactiae*, was significantly altered by the AD process. *S. agalactiae* was detected with a high prevalence in raw urban waste (0.78, 95% CI 0.45–0.94) and with a low prevalence (0.22, 95% CI 0.06–0.55) in raw agricultural OWs. The decrease in *S. agalactiae* prevalence was significant (*p* < 0.01) in digestates of the urban wastes composed mainly of WWTP sludge with or without straw +/− zeolite supplementation (*n* = 9) (Figure 2 and Supplementary Table S7).

There was no significant decrease in the prevalence of virulence factors and MGEs in digestates, regardless of the OW origin (Supplementary Table S7). Similarly, no significant change in the prevalence of ARGs could be detected in the digestates derived from OWs of agricultural origin (*n* = 11). However, in the digestates of urban wastes composed mainly of WWTP sludge (*n* = 9), the prevalence of three ARGs (*oqxA*, *bla*_CTX-M_, and *vanA*) was significantly reduced (*p* < 0.05, Supplementary Table S7).

#### 3.3.3 Effect of anaerobic digestion on absolute concentrations of sanitary indicators

**Figure 3:**
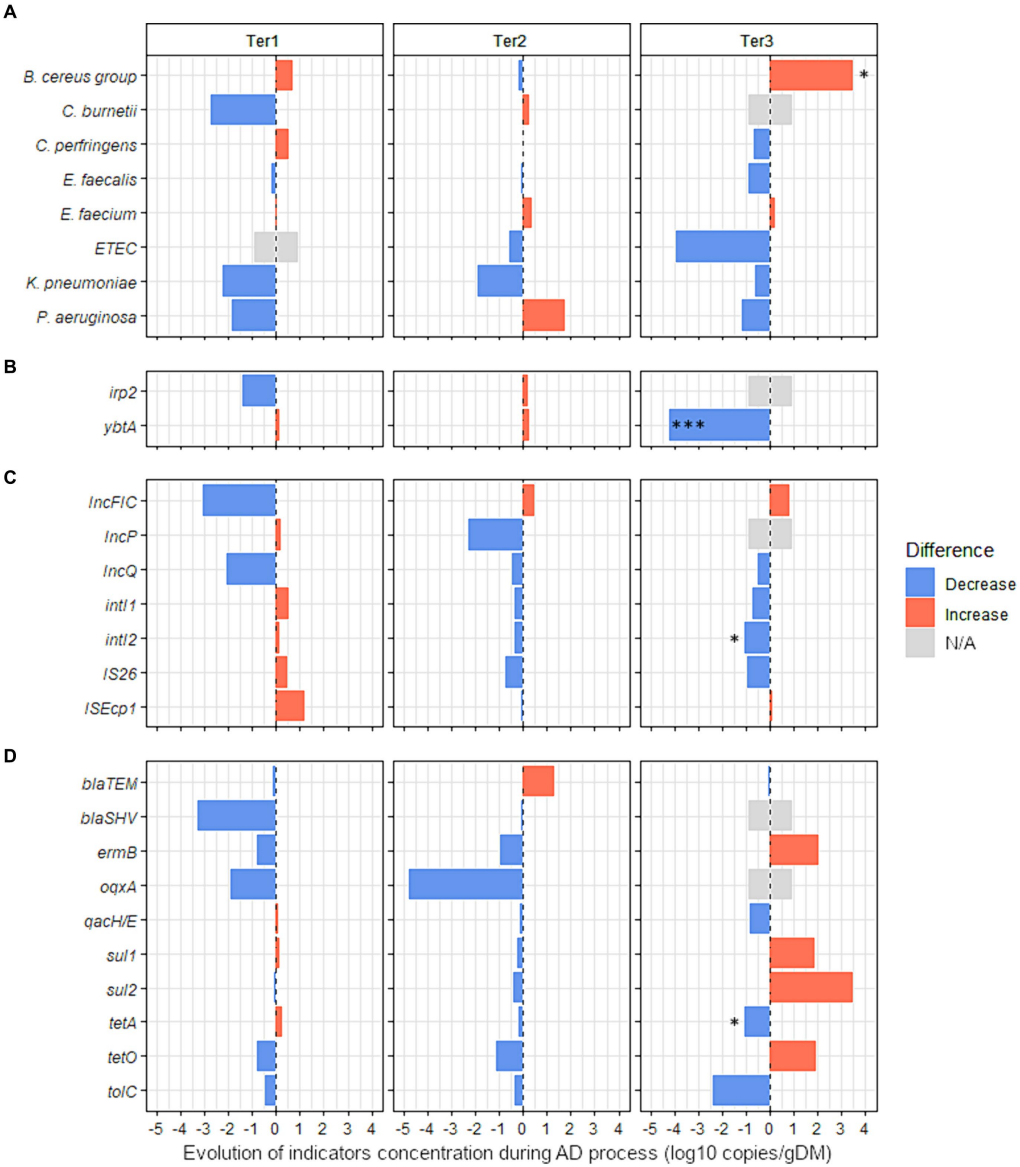
Effect of anaerobic digestion processes on the evolution of 27 sanitary indicators in organic wastes from agricultural origin. The absolute abundance of each sanitary indicator was evaluated by Droplet Digital PCR in the digestates and compared with their quantities in the corresponding raw organic wastes (see Supplementary Table S8 for more details). The effect of the key parameters of process (feeding mode, number of steps, temperature, pH) was studied using three full-scale anaerobic digestion plants (Ter1, Ter2, and Ter3) (see Table 1 for more information on operating parameters of these reactors). Blue bars represent positive effects, and red bars represent negative effects. The significance of the effect was investigated by fitting GLM with a gamma likelihood. *p*-values were adjusted using Bonferroni’s Holm method (*p* < 0.05*; *p* < 0.01**; *p* < 0.001***). N/A: Indicator non-quantifiable by ddPCR because of either a low prevalence of the indicator or an amplification of several nonspecific products by RT-PCR (see supplementary Table S8 for more information). The sanitary indicators were classified into 4 categories: **(A)** bacterial genera or species, **(B)** virulence factors genes, **(C)** mobile genetic elements, and **(D)** antimicrobial resistance genes (see Table 2 for more information on these indicators genes).

**Figure 4:**
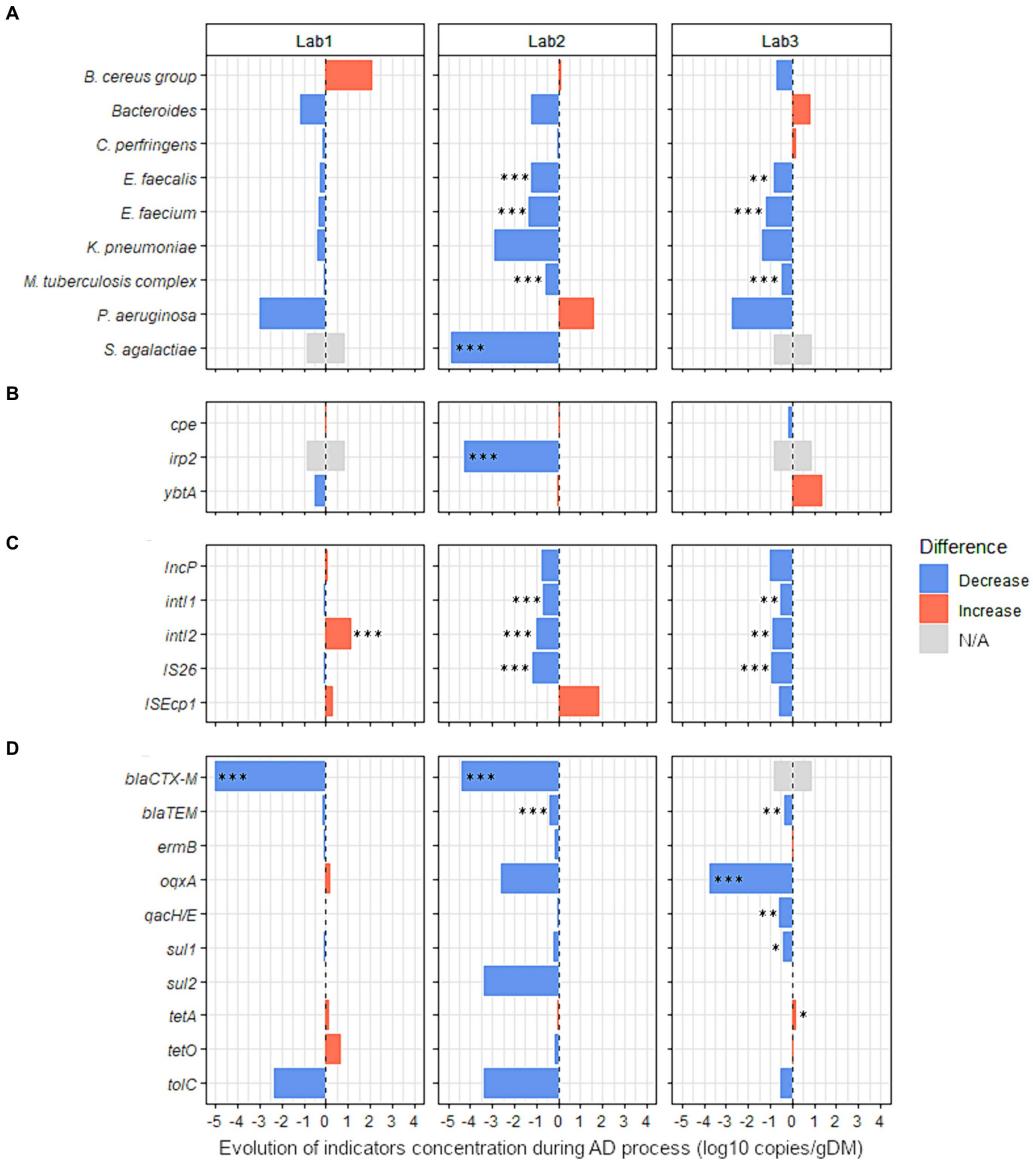
Effect of the nature of raw organic wastes on the evolution of 27 sanitary indicators during anaerobic digestion process. The absolute abundance of each sanitary indicator was evaluated by Droplet Digital PCR in the digestates and compared with their quantities in the corresponding raw organic wastes (see Supplementary Table S9 for more details). Three lab-scale mesophilic reactors (Lab1, Lab2, and Lab3) were used where the anaerobic digestion process was similar, but the proportions of inputs were varied (see Table 1 for more information on operating parameters of anaerobic reactors Lab1, Lab2, and Lab3). Lab1 was fed with 97% activated sludge (urban waste) and 3% cow manure. Lab2 was fed with 85% of the same mixture, supplemented with 15% wheat straw. Lab3 was fed with 81% of the same mixture, 15% wheat straw, and 4% zeolite powder. Blue bars represent positive effects, and red bars represent negative effects. The significance of the effect was investigated by fitting GLM with a gamma likelihood. *p*-values were adjusted using Bonferroni’s Holm method (*p* < 0.05*; *p* < 0.01**; *p* < 0.001***). N/A: Indicator non-quantifiable by ddPCR because of either a low prevalence of the indicator or an amplification of several nonspecific products by RT-PCR (see Supplementary Table S9 for more information). The sanitary indicators were classified into 4 categories: **(A)** bacterial genera or species, **(B)** virulence factors genes, **(C)** mobile genetic elements, and **(D)** antimicrobial resistance genes (see Table 2 for more information on these indicators genes).

We then used ddPCR to quantify the 31 sanitary indicators detected in more than 33% of raw samples from agricultural or urban origin and persistent in digestates (Figures 3, 4 and SupplementaryTables S8, S9). AD did not significantly alter the total bacterial abundance (16S rRNA gene) in any of the reactors (Supplementary Tables S8, S9). The average bacterial abundance was 10.0 ± 0.6 log10 copies/g in raw wastes and 10.2 ± 0.7 log10 copies/g in digestates from full-scale reactors (Supplementary Table S8). Similarly, the average bacterial abundance was 10.5 ± 0.5 log10 copies/g in the raw wastes composed mainly of WWTP sludge and 10.7 ± 0.3 log10 copies/g in the corresponding digestates obtained from laboratory-scale reactors (Supplementary Table S9). Therefore, the sanitary indicator specific gene count is expressed in log10 copies/g of dry matter, without normalization to 16S rRNA gene copy numbers.

##### 3.3.3.1 Influence of different technological processes

Three full-scale reactors with different technologies (Ter1, Ter2, and Ter3) were used to study the impact of different AD processes on the levels of 27 sanitary indicators in OWs from agricultural origin. First, the impact of AD on the absolute concentration of sanitary indicators in OWs was measured without differentiating between the different reactors (Supplementary Table S8). Of the 27 quantified indicators in raw OWs and corresponding digestates from Ter1, Ter2, and Ter3, only *oqxA* was significantly changed (−3.3 log10 gene copies/g, *p* < 0.05) by AD.

Second, raw OWs and digestates from each reactor were compared independently to identify the hygienic effect specific to each technological process (Figure 3 and Supplementary Table S8). No significant change between inputs and outputs was observed in Ter1 (two-stage, semi-continuous feeding, 44.5°C) or Ter2 (single-stage, semi-continuous feeding, 39°C). In the Ter3 reactor (batch, 45°C), a significant enrichment of 3.5 log10 copies/g was observed for the *B. cereus* group (*p* < 0.05, Figure 3 and Supplementary Table S8). In contrast, the virulence associated gene *ybtA* was completely removed by AD in this reactor, with a reduction of −4.2 log10 gene copies/g (*p* < 0.001, Figure 3). Similarly, the MGE *intI2* was less abundant in the digestate than in raw OWs, with a reduction of 1.1 log10 copies/g (*p* < 0.05, Figure 3 and Supplementary Table S8). The ARG *tetA* was also less abundant (*p* < 0.05) in digestate (6.6 log10 copies/g) than in raw OWs (7.7 log10 copies/g).

##### 3.3.3.2 Influence of the type of raw waste

Three laboratory-scale reactors fed with feces with or without wheat straw and/or zeolite powder supplementation were used to investigate the effect of the type of waste input on the level of sanitary indicators in digestates. The effect of AD (single-stage, semi-continuous feeding, 37°C), was first studied without considering the nature of the added waste. Sanitary indicators were quantified and compared between raw wastes and digestates from the three studied reactors (Supplementary Table S9). *S. agalactiae* was totally removed (−4.9 log10 copies/g, *p* < 0.001) by AD and the concentration of three other species were also significantly reduced: *E. faecalis* (−0.8 log10 copies/g, *p* < 0.001); *E. faecium* (−1.0 log10 copies/g, *p* < 0.001); and *M. tuberculosis* complex (−0.4 log10 copies/g, *p* < 0.01). In addition, the concentration of three MGEs was also significantly reduced by AD: *intI1* (−0.5 log10 copies/g, *p* < 0.05); IS*26* (−0.7 log10 copies/g, *p* < 0.001); and IncP (−0.6 log10 copies/g, *p* < 0.05). Significant reductions were also noted for the ARGs *bla*_CTX-M_ (total removal of 4.7 log10 copies/g, *p* < 0.001) and *bla*_TEM_ (−0.3 log10 copies/g, *p* < 0.05).

Then, with the aim of identifying the potential variations related to the nature of waste used, the raw OWs and digestates were compared for each reactor independently (Lab1, Lab2, and Lab3, Figure 4 and Supplementary Table S9). No sanitary indicators were enriched in the digestates from Lab2. In digestates from Lab3, only *tetA* was moderately increased (0.2 log10 copies/g, *p* < 0.05). In both reactors where straw was added (Lab2 and Lab3), seven indicators were significantly less abundant in digestates than in raw OWs (Figure 4 and Supplementary Table S9). *E. faecium* was reduced by 1.4 and 1.2 log10 copies/g (*p* < 0.001) and *E. faecalis* was reduced by 1.2 and 0.8 log10 copies/g (*p* < 0.01) in Lab2 and Lab3, respectively. The *M. tuberculosis* complex also decreased by 0.6 and 0.5 log10 copies/g (*p* < 0.001) in Lab2 and Lab3, respectively. Three MGEs (*intI1*, *intI2*, and IS*26*) were also reduced in the digestates of both reactors Lab2 and Lab3 (Figure 4 and Supplementary Table S9). The *intI1* gene was reduced by 0.7 and 0.5 log10 copies/g (*p* < 0.01) and *intI2* was reduced by 1.0 and 0.9 log10 copies/g (*p* < 0.01) in Lab2 and Lab3, respectively. IS*26* decreased by 1.1 and 1.0 log10 copies/g (*p* < 0.001) in Lab2 and Lab3, respectively. Also, *bla*_TEM_ was decreased by 0.4 log10 copies/g (*p* < 0.001) and 0.3 log10 copies/g (*p* < 0.01) in Lab2 and Lab3, respectively. Similarly, in Lab2, *S. agalactiae* and *irp2* were significantly less abundant in digestates than in raw OWs, with a reduction of 4.9 and 4.3 log10 copies/g, respectively (*p* < 0.001, Figure 4, and Supplementary Table S9). In Lab3, fed with feces supplemented with wheat straw and zeolite powder, an MGE (IncP) and three ARGs (*qacH/E*, *oqxA*, and *sul1*) were decreased by −1.0, −0.6, −3.8, and − 0.4 log10 copies/g (*p* < 0.05), respectively, in digestates, compared with raw OWs (Figure 4 and Supplementary Table S9). Moreover, the beta-lactamin resistance gene *bla*_CTX-M_ was reduced by 4.4 log10 copies/g (*p* < 0.001) in Lab2 digestates. A similar effect was also observed (−5.0 log10 copies/g; *p* < 0.001) in digestates obtained from Lab1 (Figure 4 and Supplementary Table S9). However, contrary to what we observed in Lab2 and Lab3, *intI2* was increased by 1.2 log10 copies/g (*p* < 0.001) in the digestates obtained from Lab1 (Figure 4 and Supplementary Table S9).

## 4 Discussion

In France, raw OWs from different origins (agricultural or urban) can be mixed in the same anaerobic reactor (Avadí et al., 2022). Depending on their origin, these OWs may be contaminated by metallic and/or organic micropollutants and by pathogenic organisms (Aigle et al., 2021). In the anaerobic environment inside a reactor, opportunistic pathogenic bacteria can be enriched, especially if they have the functional traits necessary for their survival and/or growth. The main goal of this study was to evaluate the impact of the nature of raw OWs (using three laboratory-scale reactors Lab1, Lab2, and Lab3) and different AD processes (using three full-scale reactors Ter1, Ter2, and Ter3) on the levels of sanitary indicators in digestates. First, we selected a high number of health indicators (77) that are most often found in raw OWs including new indicators of health hazards, such as virulence factors carried by MGEs. At least, one gene from the most frequently detected ARG classes in livestock waste (sulfonamides, tetracyclines, β-lactams, macrolide-lincosamid-streptogramin B and fluoroquinolone) was selected (He et al., 2020; Zalewska et al., 2021; Tian et al., 2022). Similarly, the ARGs found frequently in activated sludge (e.g., *sul1*, *sul2*, *tetA*, *tetO*, *tetC*, *qnrA*, *vanA*, *qacE*) were also selected (Redhead et al., 2020; Shin et al., 2020; Mutuku et al., 2022).

The specific detection of 40 indicators including 13 pathogenic species/genera, four virulence factors, eight MGEs, and 15 genes implicated in antimicrobial resistance were validated in raw OWs, and their digestates, from agricultural and urban origin. We then assessed whether the origin of raw OWs affects the levels of sanitary indicators. We classified the prevalence of 40 indicators in raw OWs of different origin (agricultural *vs* urban) into three groups (high, medium, and low prevalence). From these results, three different profiles were identified.

Some sanitary indicators were highly prevalent in all raw OWs tested in this study, despite their diverse origin, including 23% (3/13) of the pathogenic species/genera (*E. faecalis*, *E. faecium*, and *B. cereus* group), 86.7% (13/15) of the antimicrobial resistance genes, and 62.5% (5/8) of the MGEs analyzed. These results are consistent with earlier studies reporting that both livestock manure and sludge can be an important reservoir of ARGs (Li et al., 2015; Karkman et al., 2018). Interestingly, the high concentrations of pharmaceutical products detected in our study could explain the high prevalence of ARGs which is likely associated with selection phenomena (Hughes and Andersson, 2012; Bengtsson-Palme et al., 2018) and horizontal gene transfers (Jutkina et al., 2018). In the mixture of activated sludge and cow manure (97:3) used to feed the Lab1 (100%), Lab2 (85%), and Lab3 (81%) reactors, three pharmaceutical classes were detected at high concentrations (antibiotics>antimicrobials>anti-inflammatory drugs). Fluoroquinolones were present at the highest concentrations, reaching values of up to 3.5 mg/kg for ofloxacin and 2.6 mg/kg for ciprofloxacin. Mailler et al. (2017) found similar concentrations of fluoroquinolones in activated sludge in France (up to 6.7 mg/kg for ofloxacin and 12.8 mg/kg for ciprofloxacin). In our study, antimicrobial products were also detected with a wide concentration range, from 160 μg/kg for triclocarban to 2.1 mg/kg for triclosan. This is lower than the concentration of triclocarban in activated sludge reported in a previous study (mean of 13 mg/kg) (Mejías et al., 2021).

**Figure 5:**
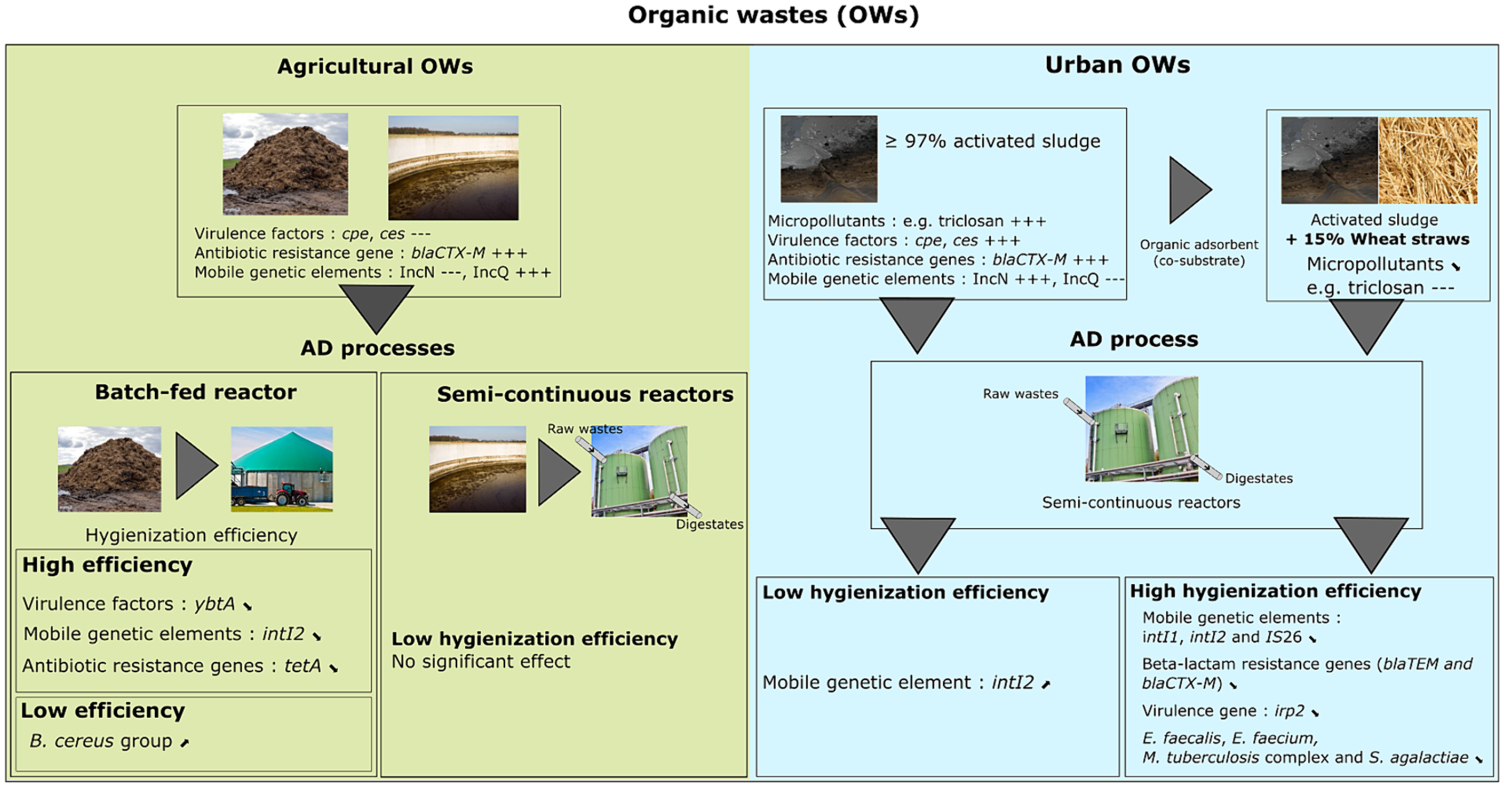
Fate and distribution of different pathogens, virulence factors, mobile genetic elements, and antibiotic resistance genes in digestates derived from different anaerobic digestion processes and various raw organic wastes.

Other sanitary indicators were only present in either agricultural or urban waste (Figure 5). While *Bacteroides* were not detected in the raw agricultural samples used in the three full-scale reactors, both *Y. enterocolitica* and enterotoxigenic *E. coli* (ETEC) were found only in raw agricultural wastes and were not detected in urban wastes. The primers used for *Bacteroides* are specific to the human HF8 cluster (Bernhard and Field, 2000), therefore, it is logical that this species complex was not found in agricultural waste. The *bla*_CTX-M_ gene was detected in urban and was not found in waste of agricultural origin. Moreover, IncQ and IncN prevalence were also associated with the OW origin because IncN plasmids were detected in urban wastes and IncQ in agricultural wastes. This is consistent with a previous study in which IncN plasmids containing a variety of antibiotic resistance (mainly streptomycin resistance) and transposition genes were isolated from a final effluent from a municipal WWTP (Eikmeyer et al., 2012). However, other authors detected IncN plasmids in animal-associated environments (Johnson et al., 2007; Moodley and Guardabassi, 2009), and therefore, we cannot exclude their potential presence in agricultural waste.

A third profile of sanitary indicators was found, for which it was not possible to identify an association between functional traits and species occurrence according to OW origin. The *cpe* and *ces* genes encoding toxins produced by *C. perfringens* and *B. cereus*, respectively, were not detected in agricultural OWs even though 44 and 78% of samples were positive for *C. perfringens* and *B. cereus*, respectively. However, in urban wastes, *cpe* was detected in 100% of tested samples (100% prevalence of *C. perfringens*) and *ces* was detected in 33% of samples (78% prevalence of *B. cereus*). Our results are consistent with earlier studies, which reported a very low prevalence of *cpe*-positive *C. perfringens* and *ces*-positive *B. cereus* in OWs of agricultural origin (Altayar and Sutherland, 2006; Cui et al., 2016; Derongs et al., 2020). In contrast, many other studies suggested that humans are a potential reservoir for *cpe*-positive *C. perfringens*, which can, therefore, be detected in urban sludge (Heikinheimo et al., 2006; Carman et al., 2008). These data, together with our results, suggest that WWTP sludge could be a selective environment for growth of *C. perfringens* and *B. cereus* carrying the *cpe* and *ces* genes and underline the importance of exploring the associations between bacterial functional traits and OW origins for a more precise assessment of the health hazards associated with OWs.

After AD treatment, the fate of sanitary indicators was also evaluated. Of the 40 sanitary indicators, only one (*S. agalactiae*) was completely removed in all digestate samples, regardless of the AD process or the nature of the raw waste. *S. agalactiae* has a better ability to form biofilms at acidic pH, whether *in vitro* or in the host vaginal tract (Ho et al., 2013; D’Urzo et al., 2014). The reactors sampled in this study operated at neutral to basic pH, which could explain the failure of *S. agalactiae* to persist during the AD process. Interestingly, a significant decrease was also observed in the prevalence of two other indicators (*bla*_CTX-M_ and *vanA*) but only in the digestate from urban origin.

As revealed by real-time PCR analysis, the prevalence of many sanitary indicators monitored in our study was similar in OWs before and after AD treatment. Therefore, to better assess the sanitary quality of digestates, we performed an absolute quantitation of 31 indicators, as well as the total bacterial abundance by ddPCR. Bacterial abundance was similar in the raw OWs from agricultural and urban origin and not impacted by any of the AD treatment processes (Ter1, Ter2, and Ter3) or by the nature of the raw wastes tested in this study (Lab1, Lab2, and Lab3). We then investigated whether the nature of the raw OWs used to feed the Lab1, Lab2, and Lab3 reactors affected the abundance of 27 sanitary indicators in the digestates. AD led to a moderate increase in *tetA* in Lab3, but no other enrichment was observed in Lab2 and Lab3. In contrast, seven sanitary indicators (*E. faecalis*, *E. faecium*, *M. tuberculosis* complex, *intI1*, *intI2*, IS*26*, and *bla*_TEM_) were significantly decreased in the digestates from the Lab2 and Lab3 reactors, where 15% wheat straw was added to the OW inputs. This was in direct contrast to Lab1, fed with urban waste without wheat straw, where *intI2* increased in digestates. These results suggest that adding wheat straw may allow a better sanitizing effect during AD of urban wastes (Figure 5). The hydraulic retention time (HRT) was extended by 20 days in Lab2 and Lab3, compared with Lab1. This is due to the lignocellulosic properties of the added wheat straws, leading to slower processing by bacterial communities within the reactors. This longer retention time may have contributed to the decrease in certain sanitary indicators observed in the Lab2 and Lab3 reactors, such as different ARGs as suggested by (Haffiez et al., 2022). Also, Song et al. (2017) showed that mesophilic reactors using 30% straw in addition to pig manure allowed the reduction of ARGs by 4.23 log (Song et al., 2017). Furthermore, cellulose-based materials are often used to adsorb residues of antibiotics, heavy metals, and organic pollutants in wastewater treatment (Hokkanen et al., 2014; Jamshaid et al., 2017; Lu et al., 2021). A similar effect was observed in our study. We found that 11 PHPs and 11 PAHs were more concentrated in the reactor without wheat straw (Lab1), compared with the reactors (Lab2 and Lab3) supplemented with wheat straw. Many studies have reported the effects of various parameters (e.g., solids content, surface area, pH, temperature, and physico-chemical properties of the different micropollutants) on the biosorption processes within a solid matrix (Rathod et al., 2015; Semblante et al., 2015; Madariaga-Segovia et al., 2023). In our conditions (pH = 7.3, temperature = 37.5°C), the physicochemical characteristics of micropollutants can mainly influence their partition between the aqueous and particulate phase. For example, the high hydrophobicity of many compounds such as triclosan have an impact on their interactions with a solid matrix. However, other PHPs such as tetracyclines and fluoroquinolones have polar functional groups and can highly adsorb onto organic matter via electrostatic interactions regardless of their hydrophobic nature. As sub-inhibitory concentrations of antibiotics and heavy metals are known to promote horizontal gene transfer (Jutkina et al., 2018; Zhang Y. et al., 2018), the adsorption of these molecules during the AD process could reduce their bioavailability. This may lower the selection pressure linked to their concentration and, therefore, limit the dissemination of resistance associated genes. Thus, the lack of improvement in sanitary quality and the significant increase in *intI2* in Lab1 may be associated with the high concentration of organic micropollutants present in this reactor. For example, the triclosan concentration in Lab1 was 2.1 mg/kg of dry sample, compared with 0 mg/kg and 0.5 mg/kg in Lab2 and Lab3, respectively. Triclosan concentrations between 0.02 μg/L and 2 mg/L (e.g., concentrations found in urban sludge or wastewater) can promote the dissemination of certain ARGs, such as *tetA*, *tetR*, and *aphA*, via horizontal transfer of the plasmid carrying these genes (Lu et al., 2018). A high triclosan concentration can lead to increased levels of reactive oxygen species (ROS), favoring an increase in the permeability of cell membranes and promoting horizontal transfer. In a more recent study, Lu et al. (2022) showed that exposure of the aquaculture pathogen *Edwardsiella piscicida* to triclosan concentrations between 2 and 20 μg/L promoted conjugative transfer among donor and *E. coli* recipient strains (Lu et al., 2022).

Some sanitary indicators, including an MGE (IncP) and three ARGs (*qacH/E*, *oqxA*, and *sul1*) were significantly decreased after AD only if wheat straw and zeolite powder were added (Lab3). This is consistent with a previous study showing that zeolite enhanced ARG reduction during mesophilic AD of swine manure, possibly due to passivation of heavy metals by zeolite, which, unlike antibiotics, cannot be degraded during AD (Zhang et al., 2012; Zhang J. et al., 2018). As animal manures are a source of heavy metals, this passivation could lower the long-term co-selection pressure exerted by heavy metals on ARGs during AD (Zhang et al., 2012; Zhang J. et al., 2018). Another study investigated the impact of zeolite on ARGs in aquaculture wastewater. Yuan et al. (2022) showed that zeolite had a significant impact on the absolute abundance of sulfonamide resistance genes (*sul1* and *sul2*). Zeolite removed up to 90% of sulfonamide ARGs, which the authors suggested was due to altered microbial community structure and abundance (Yuan et al., 2022). Many other studies have reported that 16S rRNA and ARG copy numbers are positively correlated (Yi et al., 2017; Song et al., 2018). In our study, the effect of AD on ARG copy numbers could not be correlated to a decrease in total bacterial abundance. We saw no decrease in total bacterial abundance in digestates, regardless of the type of AD process or the nature of the raw wastes.

We also investigated the impact of different AD processes on the abundance of sanitary indicators. None of the 27 indicators monitored in OWs of agricultural origin was significantly reduced by AD in either of the semi-continuous feeding reactors: Ter1 (two-stage, 44.5°C) and Ter2 (single-stage, 39°C). The batch feeding reactor, Ter3 (45°C), had a better hygienization effect, with complete removal of *ybtA* and a significant reduction in *intI2* and *tetO*. *ybt* is part of the genetic island called the High Pathogenicity Island (HPI) and is involved in regulation of siderophore-mediated iron acquisition (Yersiniabactin) (Carniel, 2001). Virulence HPI genes are essential for expression of the virulence phenotype (Carniel et al., 1987, 1989). Two hypotheses may explain the reduction of the *ybtA* gene. First, in a basic reactor such as Ter3 (pH = 9), the microorganisms will undergo very high selection pressure and only those which possess the most effective siderophores with a very strong affinity to chelate traces of ferric iron will be able to survive. Second, in an environment characterized by the probable presence of certain antibiotic residues (livestock waste), the production of siderophores is unfavorable for the survival of bacteria. The first hypothesis is based on the fact that bacteria undergo ferric stress in a basic medium (pH = 9) where ferric iron is less soluble mainly in the form of ferric hydroxide (Fe(OH)_3_) (Boukhalfa and Crumbliss, 2002; Hider and Kong, 2010; Caza and Kronstad, 2013). Therefore, only bacteria producing siderophores with a very strong affinity that can chelate traces of ferric iron Fe^3+^ can survive. The second hypothesis is based on the work of Braun (1999), Mislin and Schalk (2014), and Pramanik and Braun (2006), which showed that iron acquisition pathways using siderophores allow the internalization of certain antibiotic molecules, becoming a disadvantage for bacteria (Braun, 1999; Pramanik and Braun, 2006; Mislin and Schalk, 2014). However, despite the hygienization effect observed in this reactor, a significant enrichment of 3.5 log10 copies/g was observed for the *B. cereus* group. The presence of *B. cereus* is desirable in agricultural soil due to their ability to fix nitrogen (Iniguez et al., 2004; Yousuf et al., 2017) but their enrichment in digestates, which are usually spread on agricultural land, could pose a health problem through the food chain.

In conclusion, our data suggest that the mesophilic AD process may not fully eliminate major pathogenic bacteria and associated virulence and resistance genes, and that pollutants may interfere with hygienization. Furthermore, adding co-substrate, such as wheat straw may overcome the detrimental effect of pollutants (Figure 5). To our knowledge, this study represents the most complete survey of sanitary indicators of public health concern in digestates derived from various raw OWs and anaerobic digestion processes in France. Based on the One Health concept as defined by the World Health Organization (WHO), we chose indicators that address health risks at the animal-human-ecosystems interface, including: (1) chemical products (or residues) linked to human activity, including veterinary care, (2) well-known bacterial pathogens including, but not limited to, zoonotic agents, and (3) indirect health indicators such as ARG and MGE, which are considered gold standard indicators for studies of global health concern. These latter were selected based on the most frequently detected ARG classes in organic wastes of agricultural and/or urban origin (He et al., 2020; Mutuku et al., 2022). Nevertheless, our study has some limitations: (1) some ARGs (*sugE*, *pepA*, *qnrB*, *qacA*, *qnrA*, *fabK*, *tetM* and *vanB*), initially selected to be monitored in our study, could not be specifically amplified in the OWs samples from agricultural and urban origin, and (2) another limitation concerns four MGEs (*intI3*, IS*cr1*, IS*100* and IncW trwAB), which could not be amplified specifically in our study. The specific amplification of each target gene was investigated by real-time PCR using primers previously published. To select the primers, we considered the number of samples used to validate these primers and the origin of samples, preferring to have organic waste as matrices if possible. Despite the precautions taken, among the primers selected for our study: (1) some were previously validated in samples of clinical origin where the microbial communities were different from the microbial communities found in organic waste; (2) some were validated in a limited number of samples, and (3) detection technologies used previously to amplify some indicators have advanced. To our knowledge, this is the first report to use the ddPCR technique to quantify so many health indicators in environmental samples. This technique has been initially used as a reliable and ultraprecise method in the diagnosis of infectious disease (Cheng et al., 2019; Yan et al., 2019; Zeng et al., 2020).

## Data availability statement

The original contributions presented in the study are included in the article/Supplementary material, further inquiries can be directed to the corresponding author.

## Author contributions

CW: Conceptualization, Data curation, Formal analysis, Methodology, Validation, Writing – original draft, Writing – review & editing. BC: Conceptualization, Validation, Writing – review & editing, Funding acquisition, Investigation, Project administration, Resources. CM: Funding acquisition, Writing – review & editing, Methodology. MB: Methodology, Writing – review & editing, Data curation, Formal analysis. LL: Data curation, Methodology, Writing – review & editing. FL: Data curation, Writing – review & editing, Funding acquisition. KF: Data curation, Writing – review & editing. NS: Data curation, Writing – review & editing, Conceptualization, Formal analysis, Methodology. C-SH: Data curation, Writing – review & editing. SH: Data curation, Writing – review & editing, Funding acquisition, Methodology. DP: Data curation, Funding acquisition, Methodology, Writing – review & editing, Conceptualization, Formal analysis, Resources. GG: Funding acquisition, Methodology, Writing – review & editing, Data curation, Project administration. WG: Data curation, Funding acquisition, Methodology, Project administration, Writing – review & editing, Conceptualization, Formal analysis, Investigation, Resources, Software, Supervision, Validation, Visualization, Writing – original draft.
